# Multiomic Sequencing Reveals Distinctive Gene Expression and Epigenetic Alterations Associated With Primary Sclerosing Cholangitis Development in Treatment-Naïve Pediatric Ulcerative Colitis

**DOI:** 10.1016/j.gastha.2024.11.002

**Published:** 2024-11-16

**Authors:** Alejandra Rodriguez-Sosa, Ololade Lawal, Ciaran McDonnell, Luke Grant, John O’Brien, Muhammad Ali, Ian Stephens, Grainne Kirwan, Flavia Genua, Alexander Kel, Anna Dominik, Roisin Stack, Gregory Yochum, Michael McDermot, Glen Doherty, Seamus Hussey, Sudipto Das

**Affiliations:** 1 Epigenetics of Gastrointestinal Diseases Research Group, School of Pharmacy and Biomolecular Sciences, Royal College of Surgeons in Ireland, Dublin, Ireland; 2 Department of Pathology, Children’s Health Ireland, Dublin, Ireland; 3 GeneXplain GmbH, Wolfenbuttel, Germany; 4 DOCHAS Study, Children’s Health Ireland, Dublin, Ireland; 5 Center for Colorectal Disease, St. Vincent’s University Hospital, University College Dublin, Dublin, Ireland; 6 Division of Colon and Rectal Surgery, Department of Surgery, College of Medicine, The Pennsylvania State University, Hershey, Pennsylvania; 7 Department of Biochemistry and Molecular Biology, College of Medicine, The Pennsylvania State University, Hershey, Pennsylvania; 8 Department of Paediatrics, UCD and Royal College of Surgeons in Ireland, Dublin, Ireland

**Keywords:** Primary Sclerosing Cholangitis, Ulcerative Colitis, Transcriptional Regulation, Methylation Profiling

## Abstract

**Background and Aims:**

Primary sclerosing cholangitis (PSC) is a progressive cholestatic disease with up to 80% of patients also suffering from ulcerative colitis (PSC-UC). The difficulty in the diagnosis along with the increased risk for developing cancer represents a clinical challenge. Furthermore, the precise molecular factors regulating the phenotype of this disease subtype remain unknown.

**Methods:**

We applied methyl-capture sequencing and mRNA sequencing to colonic mucosal biopsies from 3 groups of treatment-naïve children at diagnosis from the Determinants and Outcomes in CHildren and AdolescentS study: UC (n = 10), PSC-UC (n = 10), and healthy controls (n = 10).

**Results:**

Differential gene expression between UC and PSC-UC showed significantly higher gene expression changes in PSC-UC patients when compared to UC. Specifically, expression of these genes was regulated by master transcriptional regulators (NLRP3, DLL1) and transcription factors (RELA, Myogenin, and FOXO1), which are shown to regulate expression of inflammatory response and immune-associated genes in PSC-UC patients exclusively. Differential methylation analysis between PSC-UC and UC demonstrated >2000 differentially methylated regions with a large proportion of them enriched in gene promoter and enhancer regions. We further show no difference in epigenetic age between PSC-UC and UC. Finally, we identify KLHL17 as hypomethylated and upregulated in PSC-UC patients.

**Conclusion:**

Our study, for the first time, identifies distinct gene expression and DNA methylation alterations that differentiate UC from PSC-UC at diagnosis in treatment-naïve pediatric patients. We show the gene expression differences observed between PSC-UC and UC are modulated by intricate molecular mechanisms involving master transcriptional regulator-mediated signaling through transcription factors. These findings suggest the potential utility of these molecular markers as predictive biomarkers for PSC development in UC at an early stage of development. Further validation in larger patient cohorts is warranted.

## Graphical abstract

**Figure:**
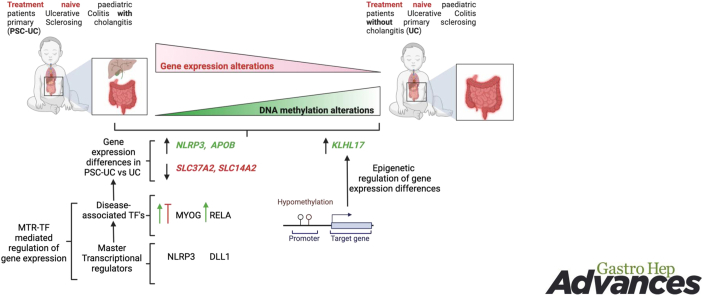

## Introduction

Primary sclerosing cholangitis (PSC) is a chronic, progressive cholestatic disease characterized by inflammation and fibrosis of the intrahepatic and/or extrahepatic ducts.^1^^,^^2^ Up to 80% of patients also have inflammatory bowel disease (IBD). These PSC and IBD (PSC-IBD) have a 3-fold higher risk of developing colorectal cancer and cholangiocarcinoma compared to patients with IBD alone.^1^^,^^3^ Early diagnosis is particularly challenging, as cholangitis is often asymptomatic until disease complications arise. PSC-IBD children are more susceptible to growth impairment than children with IBD alone.^4^ While PSC-IBD can occur in any IBD subtype ulcerative colitis (UC) is most common (80%).^5^ Atypical endoscopic features (relative rectal sparing, backwash ileitis, and predominant inflammation in the proximal colon) are more common in children with PSC-associated ulcerative colitis (PSC-UC) than classical UC and endoscopic findings are less severe.^6^

Complex inflammatory processes are orchestrated by a milieu of genetic and epigenetic alterations that regulate disease-associated gene expression patterns and consequentially lead to disease progression. Adult and pediatric patients with PSC-UC have distinctive genetic and immunological features, including a lack of overlapping single nucleotide polymorphism/risk loci between PSC-UC and non–PSC-UC.^2^^,^^7^^,^^8^ Furthermore, the persistence of activated T-memory cells is implicated in promoting hepatic inflammation in PSC-UC.^6^ Dysregulation of bile acid homeostasis and alterations in the microbiota potentially contribute to the development of PSC as an extraintestinal manifestation of UC.^6^^,^^8^ While these observations indicate multifactorial etiology, there is a limited understanding of the molecular factors underpinning the development and phenotype of PSC-UC in children.

Here, we hypothesize that transcriptomic and epigenetic differences exist between pediatric patients with UC and PSC-UC, which impact their underlying clinical phenotypes. To distinguish PSC-UC from UC, we conducted integrative multiomic analysis including DNA methylation and mRNA sequencing on mucosal biopsies from a rare and unique cohort of treatment-naïve pediatric patients with PSC-UC, UC-only, and healthy controls. Using multimodal bioinformatic pipelines, we identified unique gene expression and DNA methylation alterations in PSC-UC compared to UC and established the regulatory processes controlling these changes in pediatric patients.

## Materials and Methods

### Patient Cohort

The Determinants and Outcomes in CHildren and AdolescentS (DOCHAS) study is a prospective cohort study of all new pediatric patients with IBD diagnosed in Ireland since 2012 (ethical approval GEN/193/11). Patients are recruited before diagnosis while they are treatment-naïve and followed prospectively until discharge to adult services. Comprehensive environmental, clinical, endoscopic, histologic, radiological, laboratory and treatment data are collected. Patients undergo full evaluation and phenotyping according to the Porto criteria and Paris classification.^9^^,^^10^ Standard double reading of histological and radiological investigations is undertaken. The diagnosis of PSC is made following the identification of typical features of PSC on magnetic resonance cholangiopancreatography, endoscopic retrograde cholangiopancreatography, and/or histology following a liver biopsy. Blood and mucosal samples are taken at diagnostic endoscopies, beginning at the first (pretreatment) procedure. Clinical management follows the IBD and PSC guidelines of the time, but is at physician discretion, and endoscopy is only repeated based on clinical needs.^11^, ^12^, ^13^

### Sample Collection

Mucosal biopsies taken during endoscopy were placed immediately in RNAlater (Thermo Fisher Scientific) and stored at −80 C. Paraffin-embedded biopsies also stored. Sample blocks containing mucosal biopsies derived from the ascending colon were retrieved for each patient and 5-μm sections were generated. Sections were stained by haemotoxylin and eoisin and sites of inflammation demarcated by the pathologist. All control tissue samples derived from healthy volunteers were ascertained to have no histological inflammation.

### Nucleic Acid Extraction

Unstained 5 × 5 μm sections for each patient sample were serially deparaffinised (in xylene), followed by serial dehydration (in ethanol) and rehydration (in water) as described previously.^14^ DNA/RNA extractions were performed using the Norgen dual nucleic acid extraction formalin fixed paraffin embedded kit (Norgen Biotek Corp). As per manufacturer’s instructions, DNA was finally quantified using the Qubit high-sensitivity quantification kit and RNA was quantified using Nanodrop 2000 (Agilent Technologies Ltd).

### mRNA Sequencing and Data Analysis

RNA quality was ascertained using the AATI fragment analyzer (Agilent Technologies Ltd). RNA libraries were generated using the Kapa RNA hyperprep library preparation kit (Kapa Biosystems) and quantified using quantitative polymerase chain reaction and pooled in equimolar amounts. The balanced library pool was applied to Illumina MiSeq to confirm index balancing. Pooled libraries were sequenced on the Illumina NovaSeq 600 platform (2 × 50 bp) at the Genomics Core Facility, Queen’s University Belfast, U.K.

Data processing was done using the nf-core RNAseq pipeline (v3.0, profile: docker)^15^ and R (v4.0).(Supplementary Methods)^16^, ^17^, ^18^, ^19^, ^20^ Differentially expressed genes (DEGs) were identified using DESeq2 and involved modeling of the raw counts, using normalization factors to attribute for variations in library depth (Supplementary Methods).^21^ The DAVID gene functional classification tool was used to perform a Gene Ontology analysis allowing visualization of the enrichment of the identified DEGs in Biological Process, Molecular Function, cellular components and Kyoto Encyclopaedia of Genes and Genomes pathways.^22^

### Identification of Transcription Factor (TF) Binding Sites and Master Transcriptional Regulators (MTRs)

Transcription factor (TF) binding site analysis and master transcriptional regulator (MTR) identification were performed using the GeneXplain analysis platform (release 2020.2)^23^ for all DEGs. The *TRANSFAC* database was used to identify composite modules of TF binding motifs, assigning a positional weight matrix score to each module to predict TFs.^24^ An upstream analysis identified MTRs using TFs from the previous step, applying a graph search algorithm to the *TRANSPATH* database to develop a signal transduction network regulating the DEGs.^25^

### Methylation Sequence Capture Sequencing

Following extraction and quantification of dsDNA for each sample, 250 ng of dsDNA in 50 μl first sonicated using the Covaris M220 to generate 180–220 bp fragments. Fragmentation was confirmed using Bioanalyzer High-sensitivity chips (Agilent Technologies). Fragmented DNA samples were used for DNA library generation using the Kapa DNA Hyperprep kit (Kapa Biosystems) followed by bisulfite conversion with the Zymo DNA methylation lightning kit (Zymo Research), followed by polymerase chain reaction amplification. Libraries were quantified using the quantitative polymerase chain reaction-based Kapa library quantification kit (Kapa Biosystems). 500 ng of each quantified bisulfite-converted DNA library was used for sequence capture using the Roche SeqCap Epi sequence capture kit (Roche Sequencing Inc) as described previously.^14^^,^^26^ A custom-designed probe panel was designed for this study. The probes encompassed promoters (−2Kb to +500 bp relative to the transcriptional start site) and enhancer regions (FANTOM 5 database),^23^ promoters associated with both protein-coding and noncoding genes such as miRNAs^24^^,^^25^^,^^27^ and lncRNAs^28^^,^^29^ previously associated with UC.^30^^,^^31^ In addition, all known CpG islands, shores and shelves from the human genome built hg19 were also included in this design. The captured library was quantified, index balanced and sequenced on the Illumina NovaSeq 600 platform (2 × 150 bp) at the Genomics Core Facility, Queen’s University Belfast, U.K.

### Analysis of Methylcapture Sequencing Data

The nf-core/methylseq pipeline (v2.3.0) was used to process and analyze the methylation sequence capture data. (Supplementary Methods) Differential methylation analysis was conducted using a combination of the R packages RnBeads (v3.16) and DMRcate (v2.14.1) to identify differentially methylated regions (DMRs). Principal component analysis was performed to assess sample clustering. Comparisons were performed between PSC-UC vs controls, UC vs controls, and PSC-UC vs UC. Significant DMRs were defined using Stouffer scores, with regions classified as hypermethylated or hypomethylated. Annotation of DMRs was done using DMRcate and Annotatr (v1.26.0). We also performed integration analysis of differential gene expression and differential methylation to identify genes that were both up- or downregulated and hyper- or hypomethylated.

### Epigenetic Age Analysis (epiTOC2)

The epigenetic age of the patients by group (PSC-UC, UC and control) was analyzed using the latest version of epiTOC2,^32^ using pcgtAge metric, which represents the relative estimate of biological aging by measuring cumulative methylation changes at specific CpG sites,^32^ reflecting the number of stem cell divisions and accumulation of replication errors—not a direct age equivalent. A higher *pcgtAge* is related to an increased number of cell divisions and potentially higher cellular stress. The distribution of the *pcgtAge* values is visualized using violin plots and an ANOVA test to identify overall differences in *pcgtAge*.

### Histopathological Analysis Using Deep Learning

A deep learning model, Cerberus,^33^ a multitask fully convolutional neural network, was used to analyze cell types in haemotoxylin and eoisin whole slide images from colonic biopsies of PSC-UC and UC patients and controls (Supplementary Methods). Preprocessed whole slide images were annotated using QuPath to exclude irrelevant regions, split into 448 × 448 pixel patches, and analyzed with Cerberus on an NVIDIA A100 GPU. Cell counts were normalized to the total number of cells, and statistical comparisons used the *Statannotations* package. Welch’s t-test with Bonferroni correction compared the means of normalized cell counts of each cell type in PSC-UC vs UC, PSC-UC vs controls, and UC vs controls.

## Results

### Demographics of the Patient Cohort

The study cohort consisted of 30 patients from the DOCHAS study, including 10 patients each with PSC-UC, UC or controls, respectively. Patient clinical characteristics, treatments and outcomes are summarized in Table. Patient matching for age, biological sex, and clinical disease activity at diagnosis was undertaken to minimize confounding factors. There was a male preponderance in all groups, and the majority of patients had pancolitis, with moderate-severe disease activity. The median segmental Ulcerative Colitis Endoscopic Index of Severity scores for the ascending colon were comparable between PSC-UC and UC.

**Table:** Clinical Characteristics, Treatments, and Outcomes

Characteristics	UC	PSC-UC	Controls
n	10	10	10
Mean age in y (±SD)	12.8 (3.3)	12.9 (3.3)	12.3 (2.8)
Male:Female	2.3:1	2.3:1	2.3:1
Paris Phenotype [n (%)]			
E1	0 (0)	1 (10)	-
E2	0 (0)	0 (0)	-
E3	1 (10)	1 (10)	-
E4	9 (90)	7 (70)	-
Unclassified	0 (0)	1 (10)	-
S0	6 (60)	5 (50)	-
S1	4 (40)	5 (50)	-
Baseline disease activity (according to PUCAI score)			
Mild	2 (20)	1 (10)	-
Moderate	5 (50)	6 (60)	-
Severe	3 (30)	3 (30)	-
Endoscopic severity per UCEIS subscore for ascending colon			
Median (25th–75th centile)	4 (3–4)	4 (1–4)	-
Laboratory results			
Raised GGT	2/7 (29)	10/10 (100)	-
Raised AST	0/10 (0)	6/10 (60)	-
Raised ALT	0/10 (0)	8/10 (80)	-
Raised alkaline phosphatase	0/10 (0)	6/10 (60)	-
Raised CRP	1/9 (11)	4/10 (40)	-
Low albumin	1/10 (10)	3/10 (30)	-
Positive pANCA	-	5/6 (83)	-
PSC characteristics			
Large duct disease	-	5 (50)	
Small duct disease	-	7 (70)	
Autoimmune hepatitis	-	3 (30)	
Fibrosis on liver histology	-	7 (70)	
Treatments used within 2 y			
5-ASA monotherapy	10 (10)	9 (90)	
Ursodeoxycholic acid	0 (0)	9 (90)	
Steroids	6 (60)	6 (60)	
Anti-TNF treatment	2 (20)	1 (10)	
Immunomodulators	3 (30)	1 (10)	
Clinical outcomes at 2 y			
Ever relapsed	3 (30)	3 (30)	
Colectomy	0 (0)	2 (20)	

5-ASA, 5-amino salicylic acid; Alk.Phos, alkaline phosphatase; ALT, alanine transaminase; anti-TNF, anti-tumor necrosis factor; AST, aspartate aminotransferase; Controls, Healthy cohort; CRP, C-reactive protein; GGT, gamma-glutamyl transpeptidase; pANCA, perinuclear antineutrophilic antibodies; PUCAI, paediatric ulcerative colitis activity index; UCEIS, ulcerative colitis endoscopic index of severity; URSO, ursodeoxycholic acid; Vit.D, Vitamin D.

### Distinctive Gene Expression Alterations Underpin Differences Between PSC-UC and UC-Only Patients

**Figure 1:**
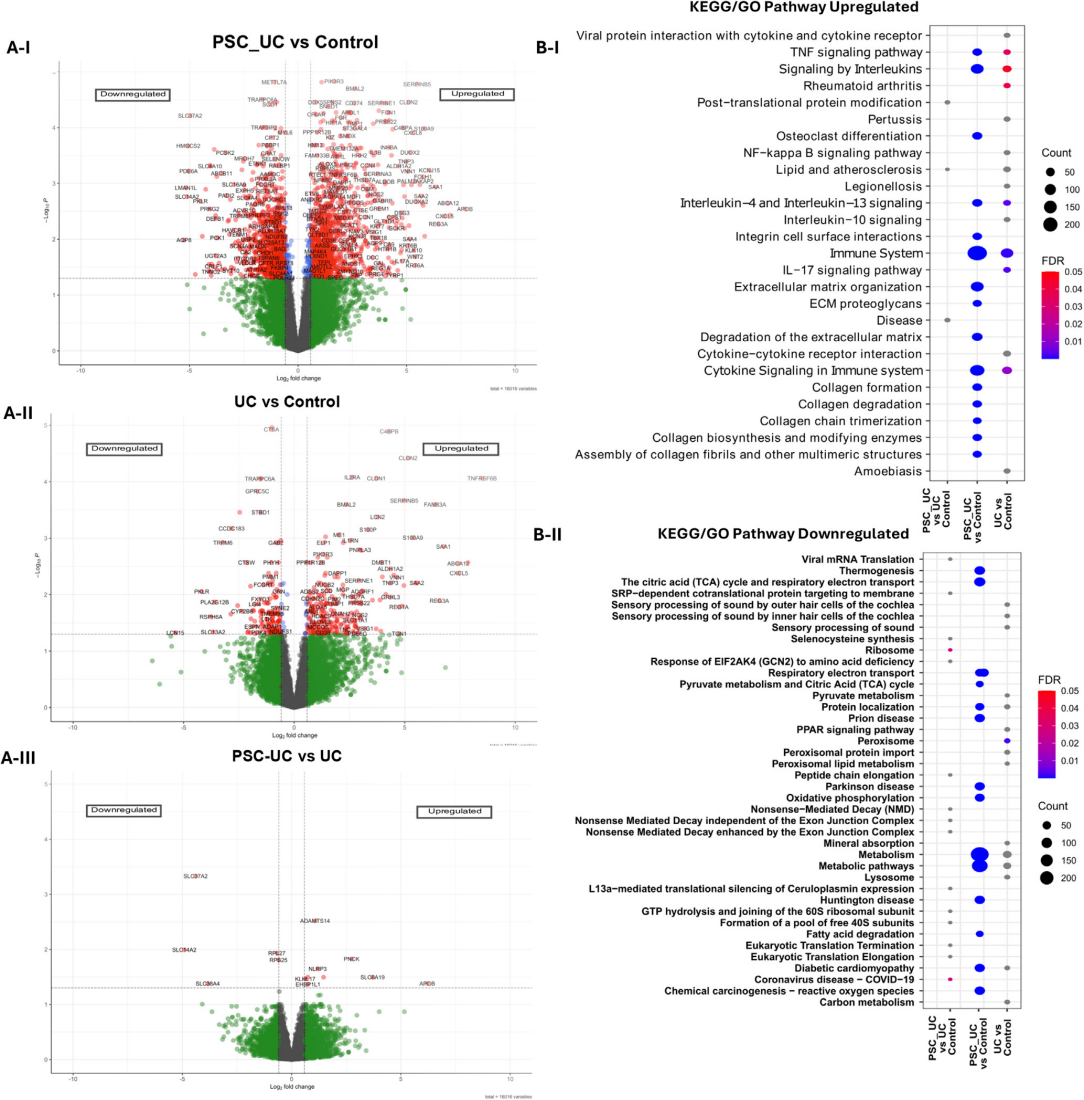
Volcano plots and Pathway enrichment analysis of differential expressed genes between each group. (A) PSC-UC versus Healthy Control (1180 upregulated and 892 downregulated), UC versus Control (249 upregulated 149 downregulated); PSC-UC versus UC (9 upregulated and 5 downregulated). Y-axis show -log10(padj) and x-axis shows FC. Cut off values of significant DEG (log2fold change ≥ 0.584 and *P*-value < .05). Significant genes are indicated in red, with upregulated represented on the right of the central axis (dotted line) and downregulated on the left of the central axis. (B) Each figure representing KEGG/GO pathways, FDR < 0.01. GO term was selected with the FDR value < 0.01 (blue to red: significant), gray (not significant). Y-axis shows pathway terms and X-axis, number of hits for each comparison: PSC-UC vs UC; PSC-UC vs control; and UC vs control. “Count”: number of genes belonging to a gene set. KEGG, Kyoto Encyclopaedia of Genes and Genomes.

Following initial quality control of the mRNAseq data (patient samples with >50% duplicate reads were excluded. Principal component analysis demonstrated an apparent separation between these PSC-UC, UC and healthy cohorts based on their transcriptomic profile (Figure A1A). This ultimately resulted in PSC-UC (n = 8), UC (n = 10) and healthy control (n = 5) being used for subsequent downstream analysis to identify DEGs (Figure A2). Differential gene expression analysis identified 1180 genes upregulated and 892 downregulated genes (p-adj <0.05) in PSC-UC patients vs controls (Figure 1A). A significantly lower number of genes were differentially expressed between UC and controls (Figure 1B), with 249 genes upregulated and 149 downregulated in UC (p-adj <0.05). We then compared PSC-UC to UC and identified 9 upregulated genes (*ADAMTS14, PNCK, NLRP3, SLC6A19, DLL1, FCGR2C, KLHL17,* Apolipoprotein B (*APOB)*, EHBP1L1) and 5 downregulated genes (*SLC37A2, SLC14A2, RPL27, RPS25, SLC38A4*) in PSC-UC (Figure 1C, Table A1).

To determine the functional characteristics and signaling pathways associated with the DEGs between the 3 clinical groups Kyoto Encyclopaedia of Genes and Genomes/REACTOME pathway analysis was undertaken. The top 3 pathways enriched for the upregulated DEGs in PSC-UC vs controls were all commonly associated with immune-related processes and included (false discovery rate [FDR] < 0.01): *Immune system pathway*; *IL-4 and IL-13 signaling* and *cytokine signaling* (Figure 1A). In contrast, top 3 signaling pathways enriched in the DEGs downregulated in PSC-UC vs control were all associated with metabolic processes and included (FDR < 0.01): *metabolism; metabolic pathways* and *TCA cycle and respiratory electron transport* and (Figure 1B). Similarly, *immune system pathway* (FDR < 0.01), *cytokine signaling in immune system* (FDR = 0.01–0.02) and *signaling by interleukins* (FDR = 0.04–0.05) (were the top 3 pathways enriched in the upregulated DEGs identified between UC vs controls (Figure 1A). In contrast, only 1 statistically significant pathway, *peroxisome* (FDR < 0.01), was identified for downregulated DEGs in UC vs controls (Figure 1B). No statistically significant pathways were identified to be enriched genes upregulated in PSC-UC when compared to UC. However, genes downregulated in PSC-UC were enriched for 2 pathways including *Ribosome* (FDR 0.04–0.05) and *COVID-19* (FDR 0.04–0.05) when compared to UC (Figure 1B).

Given our finding of an association of immune-related processes with PSC-UC and UC and previous reports of perturbed immune cell populations in PSC-IBD,^8^ we next analyzed histopathological differences between cell types, using a deep learning-based approach. The analysis revealed significant differences in neutrophil count, which was significantly higher in both PSC-UC (*P* < .01) and UC (*P* < .01) patients relative to control. Similarly, PSC-UC (*P* < .01) and UC (*P* < .01) patients had significantly higher tissue resident lymphocytes compared to controls. Furthermore, epithelial cell counts were significantly reduced in PSC-UC (*P* < .01) and UC (*P* < .01) patients relative to controls. However, we do not observe any significant differences in cell counts between PSC-UC and UC patients. Eosinophil, connective tissue cell and plasma cell counts yielded no significant differences across all comparisons (Figure A3).

### MTR and TF Mediated Signaling Networks Control Gene Expression Alterations Observed In PSC-UC and UC Patients Relative to Controls

We recently demonstrated how MTRs mediated signaling networks control gene expression changes across various disease types including cancer.^34^ Similarly, given the array of gene expression differences observed here, we hypothesized that the PSC-UC and UC specific DEGs are regulated through an MTR induced process. Therefore, we performed a systematic upstream analysis to identify core TFs and subsequently MTRs that may regulate these DEGs through signaling networks.

We identified binding sites corresponding to 378 TFs enriched in the upregulated DEGs and 338 in downregulated the DEGs when comparing PSC-UC vs control. Similarly, we identified 385 TFs in the upregulated DEGs and 342 TFs in the downregulated DEGs for UC vs control. In contrast, while binding sites for 237 TFs were enriched in the upregulated DEG’s when comparing PSC-UC vs UC, no TF binding sites were enriched for the downregulated DEGs (Table A2). Next, we sought to identify composite modules,^35^ which are TFs associated with each DEG list that work co-operatively and ultimately coregulate gene expression. To that end, when comparing PSC-UC to controls, we identified 2 composite modules: one comprising 9 TFs (RELA, Myogenin (MYOG), RARalpha (RXRA), NR4A2, RUNX1, TAL1, GCM1, VDR, and MAZ) and another comprising of 14 TFs (NMYC, NFATC1, NFATC2, MYOG, RXRA, NFATC3, SNAI2, NFATC4, CUX1, NRL, MAX, KLF4, NR1H4, and POU1F1) enriched in the upstream regions of upregulated and downregulated genes respectively.

Similarly, for DEG’s identified in UC vs healthy control comparison, we identified a composite module encompassing 12 TFs (AR, RELA, TCF3, FOSL1, CDX2, HMGA1, GLI3, HMGA2, GLI2, HDAC2, POU2F1, and GLI1) and another module with 9 TFs (PDX1, MYOG, SRF, CUX1, ESR2, NKX2-5, RELA, PAX5, and TFCP2) enriched in the upstream regions of upregulated and downregulated genes respectively. Lastly, for the PSC-UC vs UC comparison, we identified only 1 composite module encompassing 11 TFs (HSF1, STAT5A, DDIT3, FOXO1, ETV1, RELA, JUND, NR3C1, THRB, NFIB, and TFA2A) enriched in the upstream regions of upregulated genes (Table A3).

**Figure 2:**
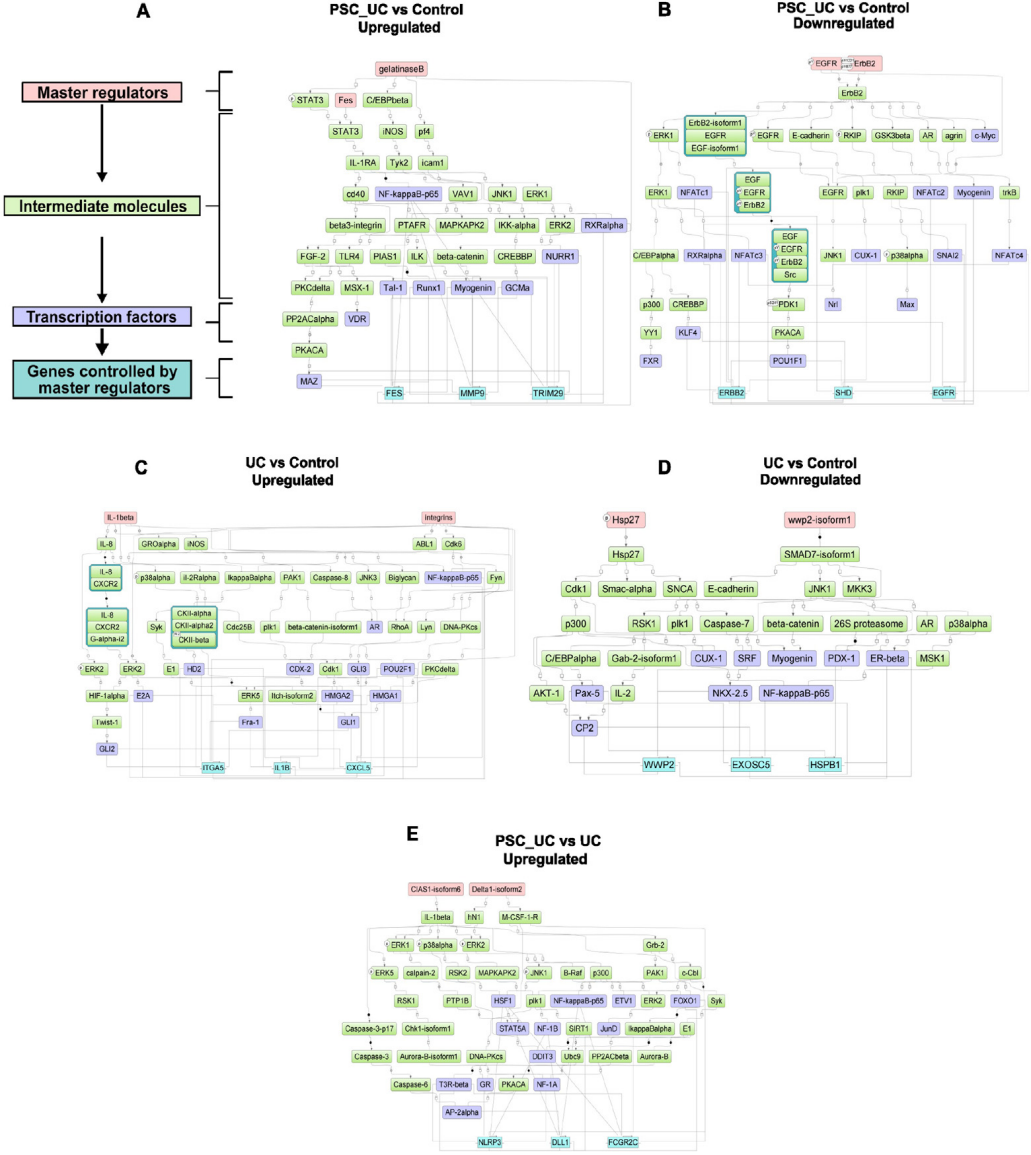
Signal transduction and gene regulatory networks examined by GeneXplain genome browser: The network diagrams were constructed using the top 2 MTRs (lowest total rank)—highlighted in pink at the top of the diagram identified as master regulators of both upregulated and downregulated genes derived from each comparative analysis: (A) Upregulated genes in PSC-UC vs healthy control; (B) Downregulated genes in PSC-UC vs healthy control; (C) Upregulated genes UC vs healthy control; (D) Downregulated genes in UC vs healthy control. (E) Upregulated genes in PSC-UC vs UC. For each comparison, the networks further illustrate the intermediate molecules (highlighted in green), TF modules (highlighted in purple) enriched for the DEGs and corresponding genes (light blue).

Next, using these composite modules, we sought to identify MTRs that regulate gene expression through positive feedback loops mediated through TFs within these modules. To that end, we have shown that MTRs (lowest *P* value) including MMP9 (Gelatinase B), FES and EGFR, ERBB2, modulate expression of upregulated and downregulated genes identified respectively in PSC-UC vs controls comparison (Figure 2A and B). Similarly, the 2 top MTRs in UC vs controls that control expression of upregulated genes included IL1B, ITGA1 (Integrins) and HSP27 and WWP2 (wwp2-isoform1) regulated expression of downregulated genes (Figure 2C and D). Finally, we have shown that top 2 MTRs regulating upregulated genes when comparing PSC-UC with UC patients included CIAS1-isoform6 (NLRP3) and Delta1-isoform2 (DLL1) (Figure 2E).

Next, we found that in addition to regulating their own expression (as shown in Figure 2), these MTRs also control expression of a diverse number of other genes. To explore this further, we first identified the top 3 TFs (from within the composite modules as described earlier) through which these MTRs exert control over gene expression, followed by identifying the specific genes regulated by these TFs from within our DEGs. To that end, we show that TFs RELA, RXRA, and MYOG commonly upregulated 350 genes in PSC-UC vs control comparison, with these genes involved in primarily “*single organism process*” and “*single-multicellular organism cellular process*” (Figure A4, Table A4). Similarly, when comparing UC vs control, a total of 57 genes, were coupregulated by TCF3, RELA, and AR, with these genes enriched for “*inflammatory response*” and pathway “*immune system*” (Figure A5).

These results together demonstrate that although TFs including RELA and MYOG are involved in regulating gene expression in both PSC-UC and UC patients; however, gene expression alterations in these patients appears to be largely regulated by MTR-TF mediated networks that are unique to each disease etiology.

### Wide-Spread Specific DNA Methylation Alterations Underpin PSC-UC and UC Phenotype Alone as Well as Differentiates the 2 Disease Etiologies

**Figure 3:**
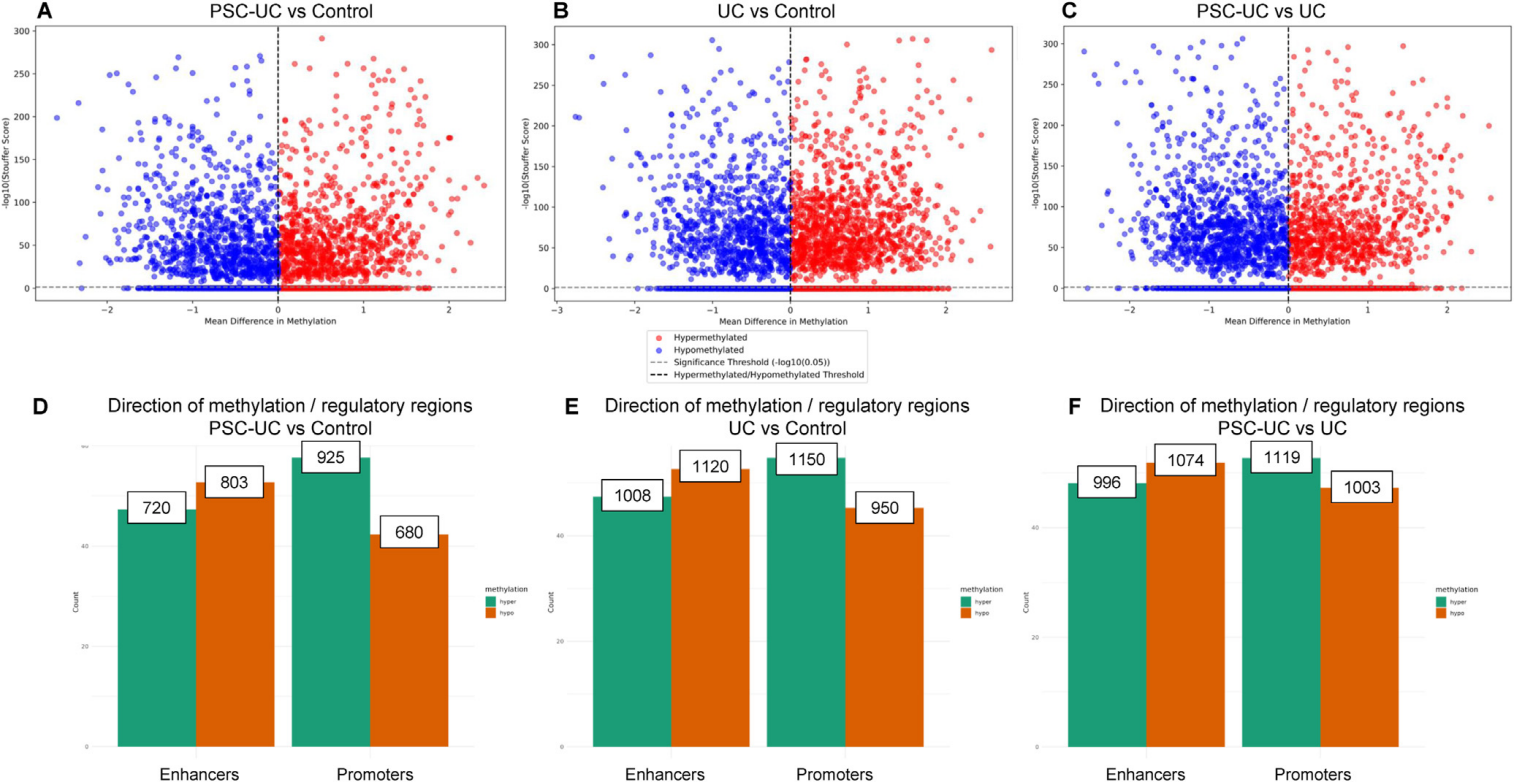
Differential methylation analysis using DMRcate. (A) PSC-UC versus healthy control (937 hypermethylated and 1026 downregulated); (B) UC versus healthy control (1241 hypermethylated and 1039 hypomethylated); (C) PSC-UC versus UC (939 hypermethylated and 1327 hypomethylated). Y-axis shows -log10(Stouffer score) and X-axis shows mean difference in methylation. Cut-off values of significant DMRs at log10 (0.05). Central axis (dotted black line) separates upregulated (red) to the right and downregulated (blue) to the left. Bar graphs shown in (D–F) highlight number (Y-axis) of hyper (in green) and hypo (in orange) methylated promoter and enhancers for each comparison.

Epigenetic alterations such as DNA methylation impact TF binding at regulatory regions, and therefore induce changes in gene expression. Following quality control 22 patients (PSC-UC = 9, UC = 8, healthy control = 5) were used for differential methylation analysis (Figures A1B and A2). The comparison between PSC-UC vs controls yielded 1972 significant DMRs, of which 937 were hypermethylated and 1026 were hypomethylated (Figure 3A). Next, comparing UC vs controls, identified 2287 significant DMRs, of which 1241 were hypermethylated and 1039 were hypomethylated (Figure 3B). The comparison between PSC-UC vs UC patients showed 2270 significant DMRs, with 939 hypermethylated and 1327 hypomethylated regions. (Figure 3C). These DMRs across all comparisons were largely localized in intergenic CpG sites followed by CpG islands (Figure A6). From a regulatory perspective we observe that while gene enhancers were largely hypomethylated, promoters however are predominantly hypermethylated across all 3 comparisons (Figure 3D–F). Furthermore, we have shown that DMRs identified between PSC-UC vs control, UC vs control, and PSC-UC vs UC were enriched at promoters mapped to 64, 59, and 9 genes, respectively. Similarly, DMRs identified between PSC-UC vs control, UC vs control, and PSC-UC vs UC were enriched at enhancer regions were mapped to 98, 104, and 13 genes, respectively.

**Figure 4:**
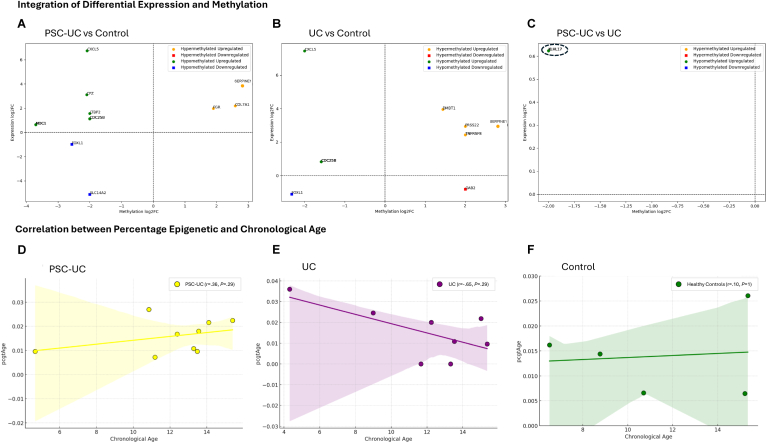
Integration of differential expression and differential methylation and correlation between epigenetic age and chronological age. Each plots (A–C) represent the genes that are both differentially methylated and expressed at the same time for each of the 3 comparisons. PSC-UC vs Control, UC vs Control, and PSC-UC vs UC respectively. The X-axis corresponds to the—log2FC(methylation) and the y-axis to log2FC(methylation). Genes identified as both significantly differentially expressed and methylated are displayed in the 4 quadrants: hypermethylated and upregulated are represented in yellow; hypermethylated downregulated in red; hypomethylated upregulated in green; and hypomethylated and downregulated in blue. Plots (D–F) represent the correlation between the epigenetic age and the chronological age for each group of patients; PSC-UC, UC, and controls. The X-axis corresponds to the chronological age and the Y-axis corresponds to pcgtAge which quantifies the rate of cellular divisions and level of biological stress.

For these genes, we compared methylation direction (hyper or hypo) with expression (upregulated or downregulated) to ascertain if expression of these genes was indeed impacted by DNA methylation ie “methylation sensitive genes.” To that end, when comparing PSC-UC with control, we identified *CXCL5, CPZ, LTBP2, CDC25B,* and *MDC1* as hypomethylated and upregulated in PSC-UC patients (Figure 4A). Similarly, *CXCL5* and *CDC25B* are both hypomethylated and upregulated, while GAB2 is hypermethylated and downregulated in UC patients when compared to controls (Figure 4B). Finally, *KLHL17* was the only gene which was identified as hypomethylated and upregulated when comparing PSC-UC with UC patients.

Previous reports assessing DNA methylation in post-treatment pediatric PSC show a significant epigenetic age acceleration in PSC-UC when compared to controls.^36^ To examine the status of epigenetic age in comparison to chronological age in our treatment naïve PSC-UC, UC, and healthy control cohorts, we applied epiTOC2 (an epigenetic clock algorithm) to methylation sequencing data derived from these 3 cohorts as described in methodology. Epigenetic age (pcgtAge values) was positively correlated with chronological age (in years) for PSC-UC (r = 0.36, *P* = .29) and controls (r = 0.10, *P* = 1) (Figure 4D and F). However, UC patients exhibited a negative correlation between epigenetic age and chronological age (r = −0.65, *P* = .29) (Figure 4E). No statistically significant difference in epigenetic age was seen when comparing all 3 groups (PSC-UC, UC, and controls) with each other (Figure A7).

## Discussion

To date, most analyses identifying PSC-UC–associated molecular alterations were performed on patient samples collected after initiation of medical treatment. Although this approach has informed our understanding of PSC-UC, it may not capture early molecular drivers of disease progression that occur before treatment. To address this, we investigated the specific molecular alterations underpinning the differences between PSC-UC and UC disease etiology, including gene expression and DNA methylation alterations, in treatment-naïve pediatric patients.

We demonstrate that upregulated genes in both PSC-UC and UC when compared to healthy control show a shared enrichment for pathways associated with the immune system and immune signaling. These immune-related signaling pathways align with our machine learning–based cell deconvolution results, which reveal an increase in lymphocytes, neutrophils, and eosinophils—all key mediators of inflammatory signaling. Furthermore, we demonstrate that enrichment of downregulated genes in metabolic pathways including TCA cycle and respiratory electron transport, was unique to PSC-UC patients. This contrasts with a recent study carried out in adult PSC-UC and UC patients, wherein genes associated with mitochondrial and energy metabolism were exclusively upregulated in UC patients.^37^ While mitochondrial dysfunction has been reported in both UC and PSC-UC patients, it is suggested that disruption of energy metabolism is likely more pronounced in PSC-UC patients owing to both liver dysfunction and systemic inflammation.^38^^,^^39^ Indeed, given our findings of such early metabolic alterations in untreated children, complimentary metabolomic studies may provide additional insights into such differences between PSC-UC and UC.

We found 14 DEGs between PSC-UC and UC patient groups. A recent study in adult patients found >1000 DEGs between PSC-UC and UC patients^8^ and it is tempting to speculate that more widespread gene expression differences may evolve over time or are impacted by treatment exposures. Furthermore, the involvement of liver in PSC-UC patients in contrast to UC patients, may also explain these gene expression differences. APOB was the most significantly upregulated gene in the PSC-UC vs UC comparison and encodes the apolipoprotein of chylomicrons and low-density lipoproteins. It has been hypothesized that accumulation of lipids in arteries—arteriosclerosis—is intrinsically linked to the accumulation of toxic biliary lipids seen in the development of PSC^40^ suggesting a potential role for *APOB* in the development and progression of PSC in UC patients. The solute carrier family (SLCs) likely impact intestinal barrier function and are implicated in Crohn’s.^41^^,^^42^ Here, we showed that 2 genes coding for solute carrier proteins—*SLC37A2* and *SLC14A2* are significantly downregulated in PSC-UC which may result in barrier dysfunction, but this requires further mechanistic interrogation. *NLRP3*—a gene from the NLR family (Pyrin Domain containing 3) which has a well-recognized role as an inducer of immunity, inflammation, and cancer development^43^^,^^44^—was upregulated in our PSC-UC group. The dysregulation of these disease-associated genes, exclusive to PSC-UC patients, can be considered a critical molecular alteration that distinguishes PSC-UC from other IBD phenotypes. Additional studies are needed to determine the precise functional and mechanistic roles of these genes in driving the PSC-UC–associated phenotype.

Gene expression changes are orchestrated through complex regulatory networks involving TFs as well as epigenetic changes. The precise regulatory mechanisms underpinning TF-mediated transcription in IBD are poorly understood.^45^ This study has identified several TFs that regulate expression of PSC-UC and UC-associated genes in treatment naïve children. For instance, TFs such as RXRA appear to upregulate expression of genes in PSC-UC patients. RXRA controls organismal development as well as homeostasis and recent reports demonstrate that RXRA regulates cellular senescence by modulating calcium signaling.^46^ Interestingly, senescence in cholangiocytes is a key driver of cholestatic disorders such as PSC, highlighting a potential mechanistic role for RXRA-mediated signaling in its pathogenesis.^40^^,^^47^ Several TFs including RELA and MYOG appear to function in a redundant manner by regulating gene expression in both PSC-UC and UC cohorts. RELA—also known as NF-Kappa-B TF p65 (NF-kappaB-p65)—has been shown to regulate intestinal inflammation through a cell autonomous Nfkb2 signaling.^48^ Our findings show that RELA, along with other TFs such as TCF3 and AR, modulates expression of genes associated with inflammatory response and immune system-related pathways. Moreover, a report assessing liver tissue following DSS-induced colitis in murine models, showed a significant increase in abundance of RELA at a mRNA level, with a further increased phosphorylation of Ser536 in the Rela protein leading to heightened function and thereby suggesting role in liver-gut cross talk.^49^

Our data provides a critical insight into the functional role of TFs in driving gene expression changes underpinning PSC-UC and UC. The TFs identified warrant further investigation to ascertain their precise mechanistic role in driving disease phenotypes and potential as a therapeutic target to impede progression in PSC-UC and UC patients.

Our bioinformatics pipeline demonstrates TF-mediated molecular changes in PSC-UC and UC, are regulated through a hierarchical signaling pathway, governed by MTRs which mediate regulatory control on genes through TFs. MTRs such as MMP9, FES, ELN, EGFR, ERBB2 and CFTR mediate wide-spread gene expression changes—including their own expression—in PSC-UC, through a positive feedback loop. In UC patients, we identified MTRs such as IL1B and ITGA1 which have previously been shown to modulate intestinal inflammation.^50^^,^^51^ Intriguingly, we show that NLRP3 and DLL1 act as an MTR which regulates expression of genes which are differentially expressed between PSC-UC and UC patients. To this end, NLRP3 has been reported to have dual functions in PSC pathogenesis, including protective function by blocking some pathways and on the other hand act as proinflammatory contributing to disease progression.^52^ Similarly, Li *et al. r*eported the expression of DLL1 to be significantly elevated in M1 macrophages in comparison with other phenotypes of macrophages. To that end, macrophages as MTRs of hepatic inflammation have been demonstrated to contribute to PSC pathogenesis^53^ and based on our MTR analysis DLL1 and NLRP3 indeed may be acting as a regulator of these macrophages or other key inflammatory components and in turn contributing towards development of PSC in the UC patients. The MTR-TF networks identified provide novel insights into the regulatory processes which drive gene expression changes and differences between PSC-UC and UC treatment-naïve pediatric patients. Pharmacological disruption of these networks presents a potential novel treatment avenue for these conditions at an early stage of disease presentation.

In addition to MTR-TF mediated gene regulation, epigenetic changes such as DNA methylation can also regulate changes in gene transcription. We observed 2270 DMRs between PSC-UC and UC biopsies. While the high number of DMRs in UC vs Control are consistent with previous reports from pediatric IBD patients,^54^ we described for the first time a higher number of DMRs (>2000) attributed to PSC-UC relative to UC, before medical treatment. The methylation differences were highly enriched in both gene promoters and enhancer regions in both PSC-UC and UC. Though the enhancer landscape in IBD was recently characterized,^55^ alterations in methylation status at enhancer regions has not been explored to date and therefore our findings prompt further investigation into the epigenetic regulation of gene enhancers on pediatric PSC-UC and UC patients and its consequential impact on gene expression.

We identified several genes in PSC-UC and UC patients whose expression is negatively correlated to direction of methylation suggesting epigenetic regulation. Specifically, when comparing PSC-UC with UC, we show that the promoter corresponding to *KLHL17* (Kelch Like Family Member 17) was significantly hypomethylated resulting in upregulation of expression in PSC-UC when compared to UC. *KLHL17* encodes for a member of the Kelch-like protein, which is involved in various cellular processes, including protein degradation through the ubiquitin-proteasome system.^56^ To date, no studies have shown a direct association of *KLHL17* with either PSC-UC or UC. However, given that protein turnover and response to cellular stress, such as inflammation, are known features of both diseases, it is likely that this gene impacts these processes.

Epigenetic age acceleration has been reported as significantly elevated in adult PSC, and those with high epigenetic age had increased prevalence of cirrhosis.^36^ In contrast, we showed that epigenetic age between healthy control, PSC-UC and UC patients are similar and demonstrate positive correlation with chronological age. Epigenetic aging is based on cell divisions resulting from biological stress, such as therapeutic interventions.^57^ The difference in our findings is likely a consequence of cohort being purely pediatric and treatment-naïve.It is thus likely that epigenetic aging in both PSC-UC and UC patients occurs at a later stage of life, or modulated by medical treatment. This study represents a robust preliminary investigation of transcriptomic and epigenetic alterations and the role of MTRs in PSC-UC and UC etiology. The limitations of this analysis include the small sample size (n = 10 per group). However, it is important to note the rarity and uniqueness of this treatment naïve patient cohort used for this study, which is also indeed comparable to other recent PSC-UC and UC-based multiomic studies.^8^ The group size was further reduced during both mRNA sequencing and DNA methylation sequencing experiments owing to the stringent quality control parameters, ultimately ensuring robustness of data.

**Figure 5:**
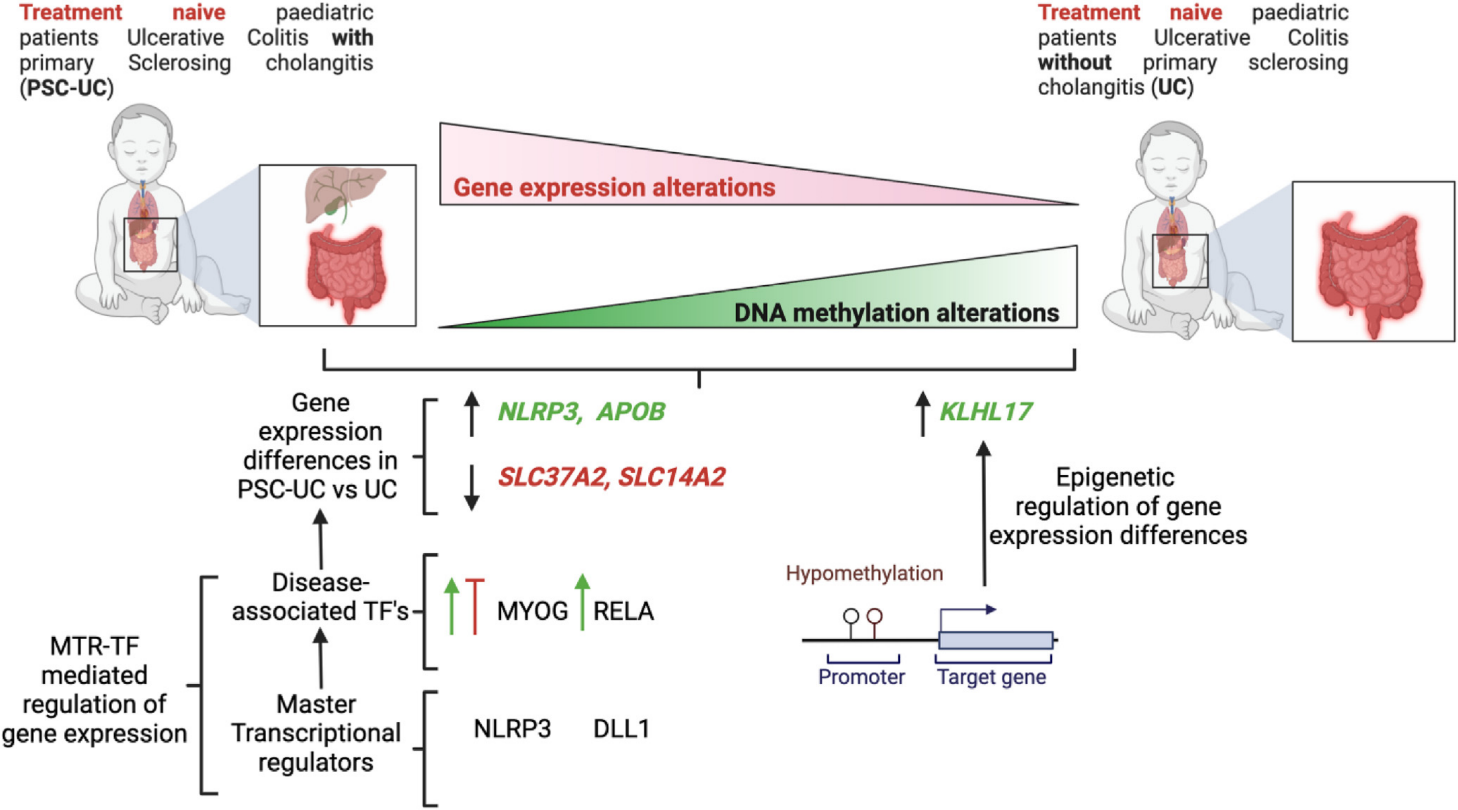
Graphical summary of the study. The diagram illustrates increased gene expression and lower DNA methylation alterations in treatment naïve PSC-UC when compared to UC patients. Two distinct regulatory mechanisms identified to modulate expression differences (upregulation or downregulation, indicated by green or red arrows) between PSC-UC and UC patients: (A) Master transcriptional regulator—TF networks, and (B) Loss of DNA methylation in PSC-UC patients can drive expression of genes such as *KLHL17*.

In conclusion, this is the first study to comprehensively describe transcriptomic and DNA methylation profiles in treatment-naïve pediatric PSC-UC and UC patients, with contextualization of responsible signaling pathways by bioinformatic analysis of MTRs and TFs that orchestrate these diseases-associated multiomic differences. These differences represent factors that may drive early PSC-UC pathogenesis (Figure 5). Indeed, this complex disease orchestration is likely mediated by an interplay between epigenetic factors, transcriptional changes and microbiome alterations^58^ associated with PSC-UC and this paves way for further studies. Nevertheless, independent validation studies are warranted to establish the potential of these differences identified in this study as predictive biomarkers for PSC-UC during childhood and/or therapeutic targets to stymy onset of this disease at an early stage of development.

## Supplementary Material

Table A1

Table A2

Table A3

Table A4

Table A5

Figure A1

Figure A2

Figure A3

Figure A4

Figure A5

Figure A6

Figure A7
