# Supplementary figures and images for: Multiomic Sequencing Reveals Distinctive Gene Expression and Epigenetic Alterations Associated With Primary Sclerosing Cholangitis Development in Treatment-Naïve Pediatric Ulcerative Colitis
Source: Gastro Hep Adv. 2024 Nov 16;4(3):100586. doi: 10.1016/j.gastha.2024.11.002 (PMC11815658; doi:10.1016/j.gastha.2024.11.002)

# Supplementary Figure 1

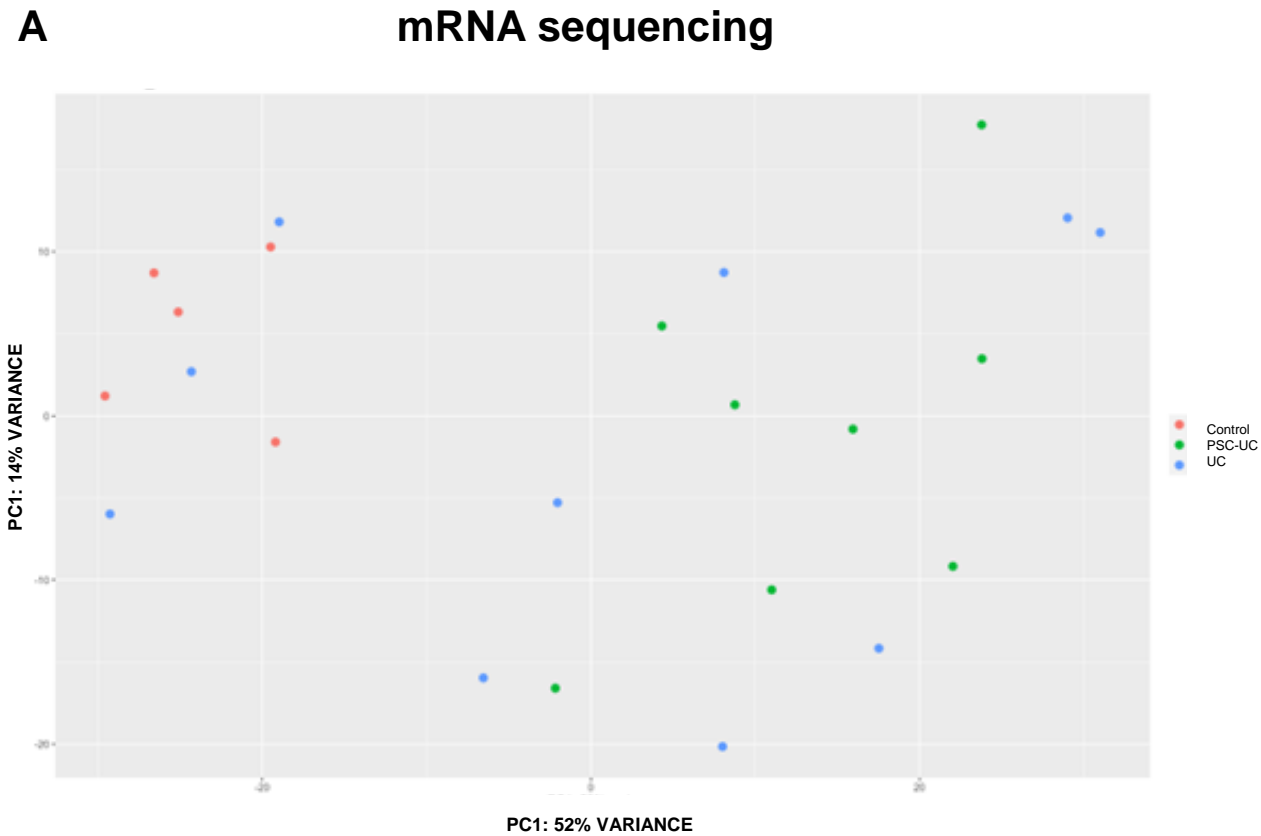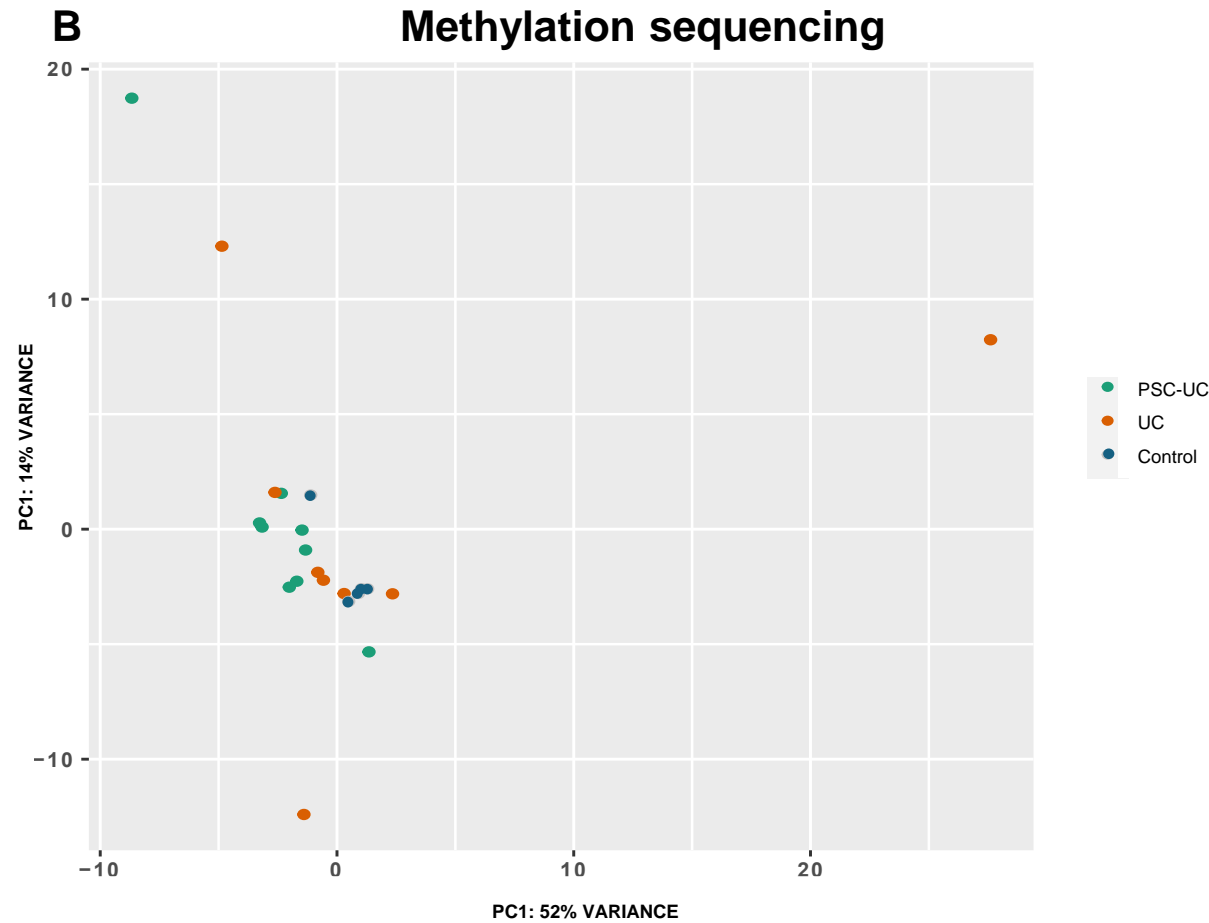

Supplement: Figure A1 [file mmc6.pdf]

# Supplementary Figure 2

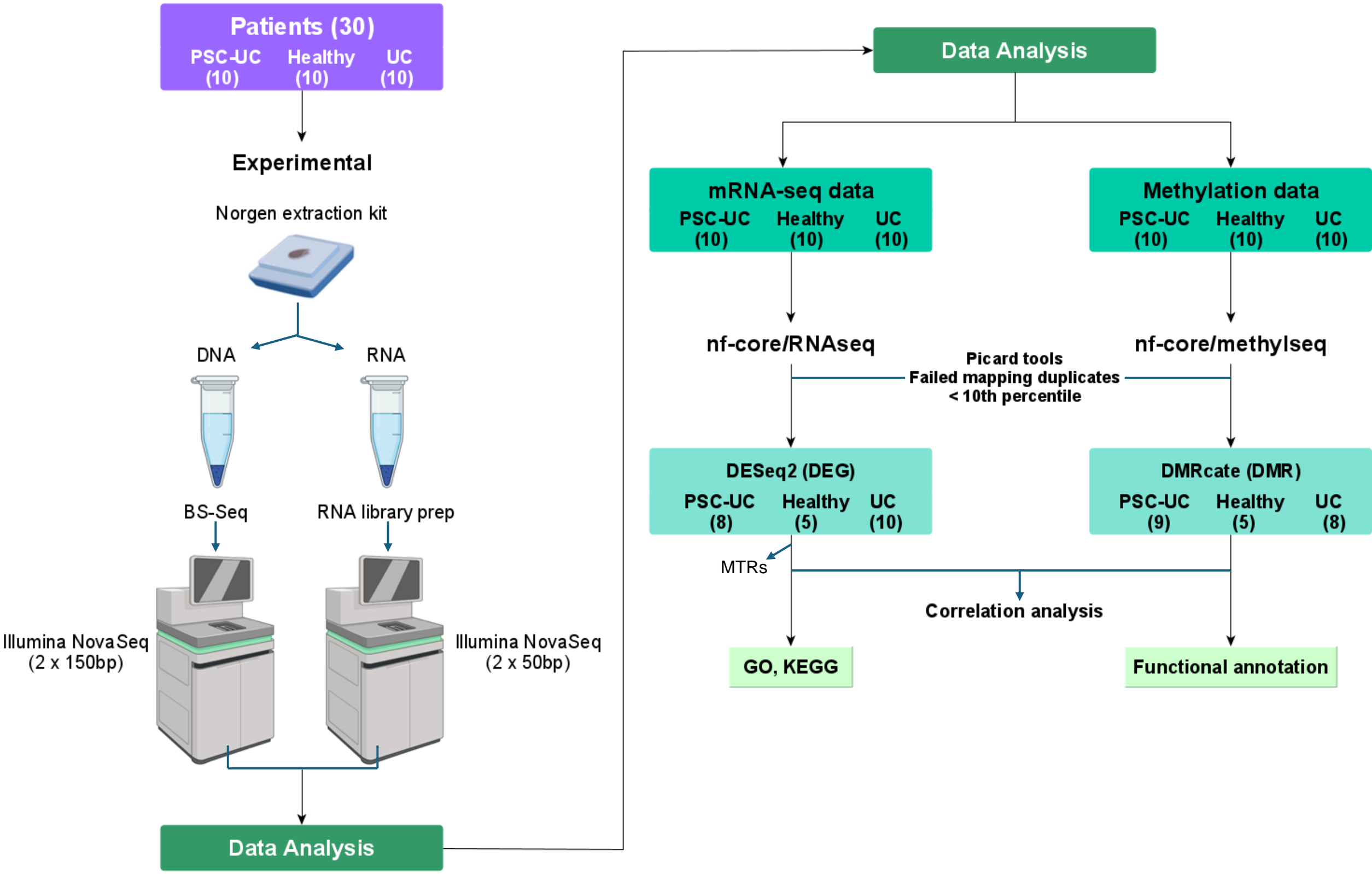

Supplement: Figure A2 [file mmc7.pdf]

# Supplementary Figure 3

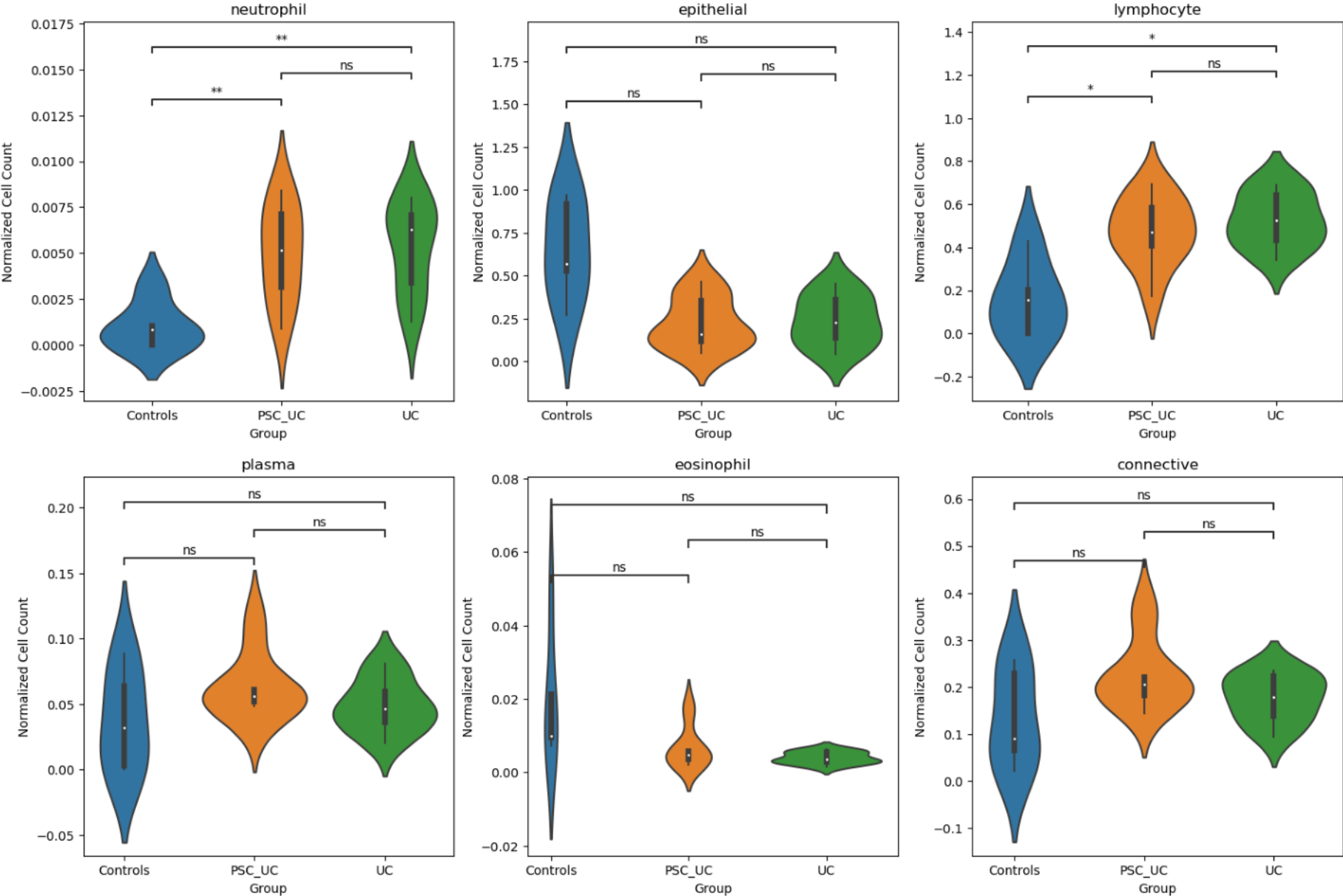

ns (not significant):  $5.00e-02 < p \leq 1.00e+00$   
\*:  $1.00e-02 < p \leq 5.00e-02$   
\*\*:  $1.00e-03 < p \leq 1.00e-02$   
\*\*\*:  $1.00e-04 < p \leq 1.00e-03$   
\*\*\*\*:  $p \leq 1.00e-04$

Supplement: Figure A3 [file mmc8.pdf]

# Supplementary Figure 4

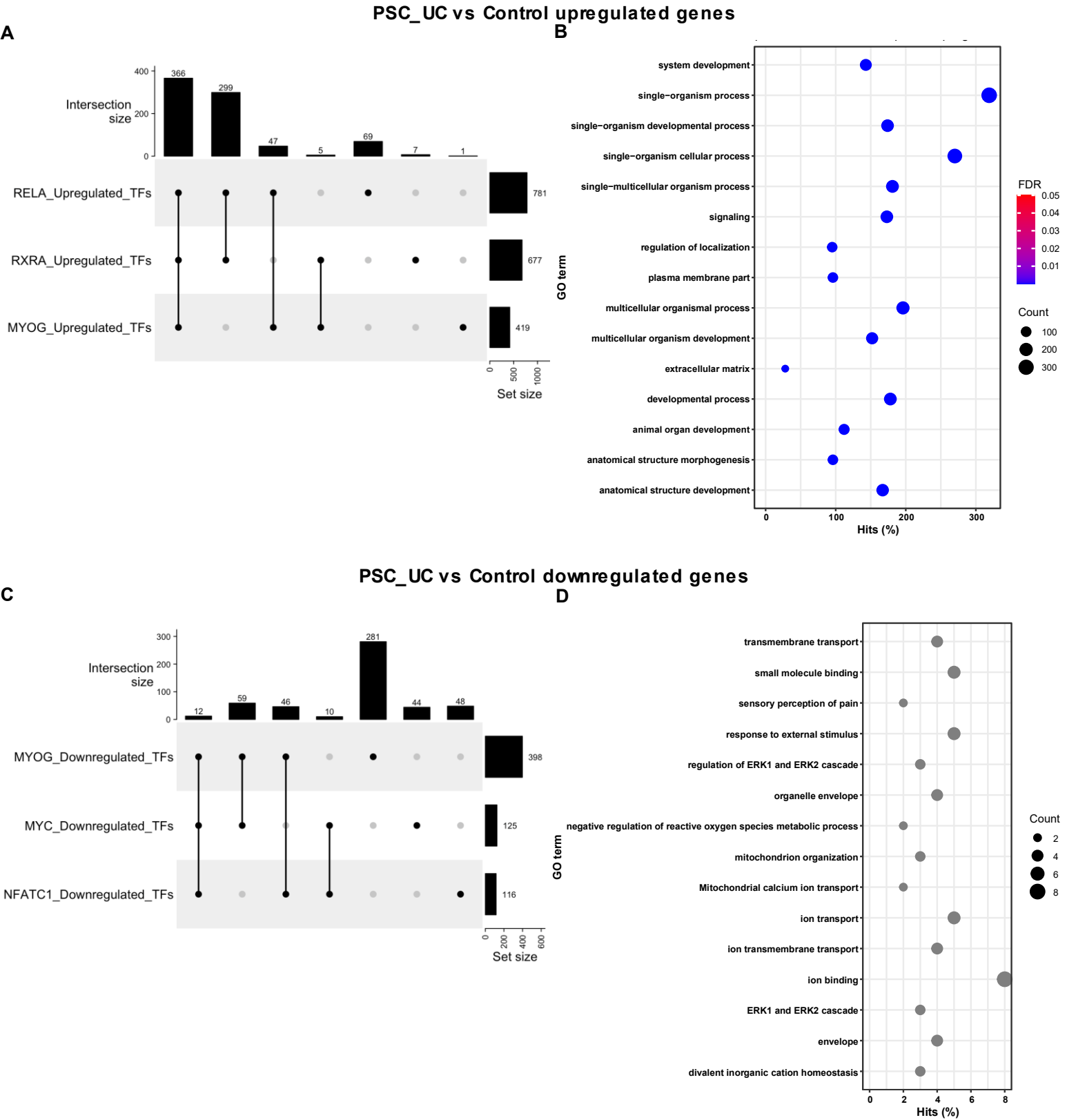

Supplement: Figure A4 [file mmc9.pdf]

# Supplementary Figure 5

## UC vs Control upregulated genes

A

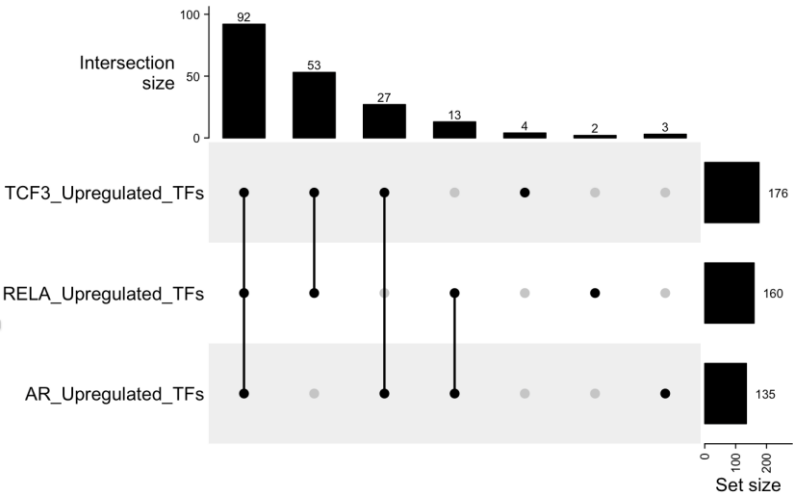

B

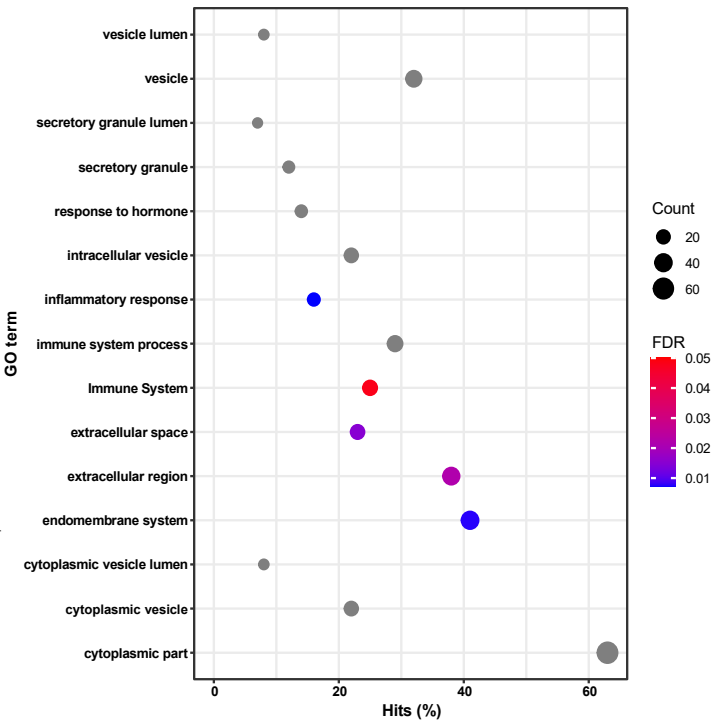

## UC vs Control downregulated genes

C

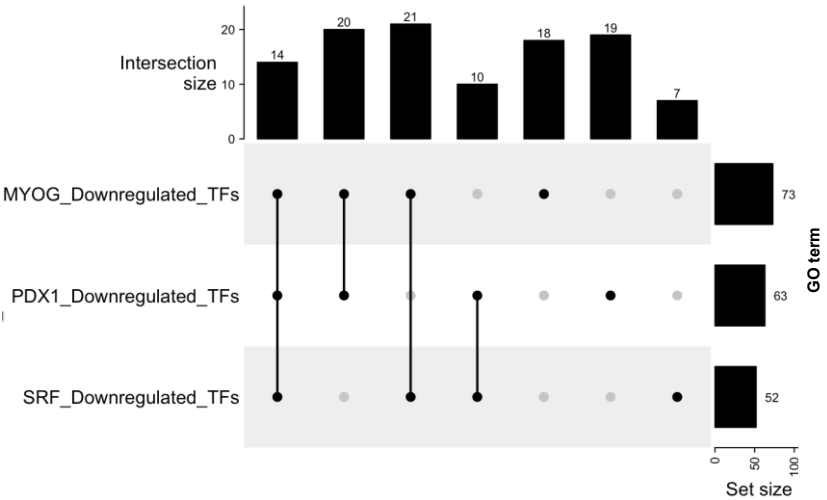

D

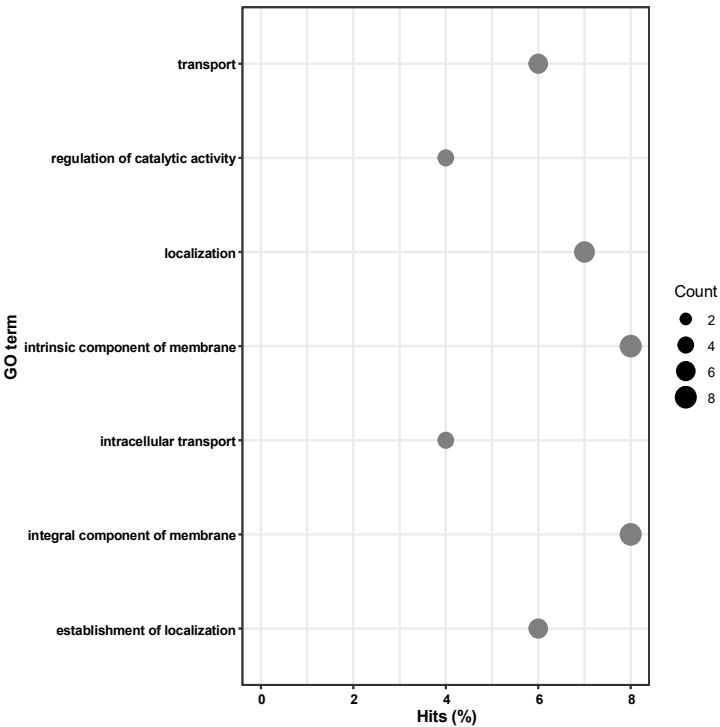

Supplement: Figure A5 [file mmc10.pdf]

# Supplementary Figure 6

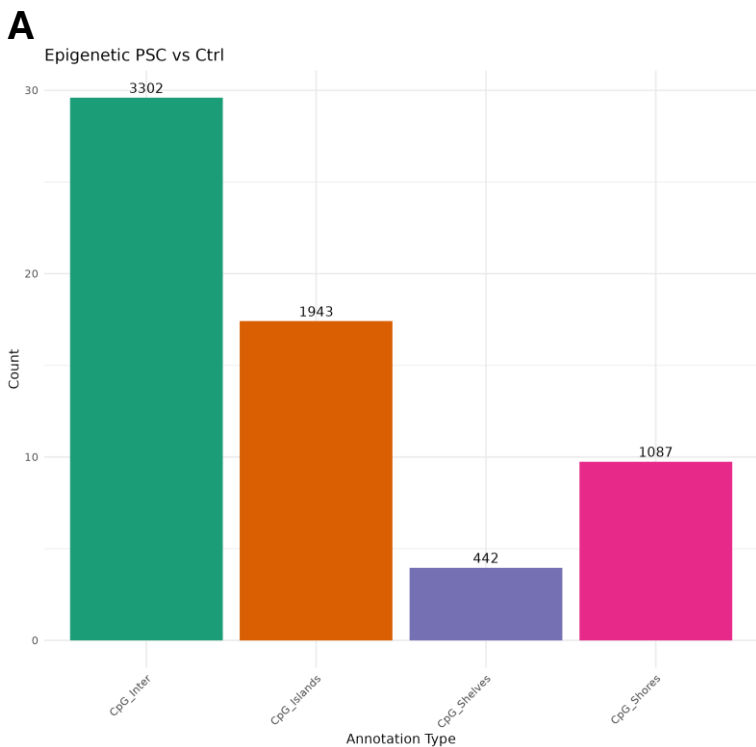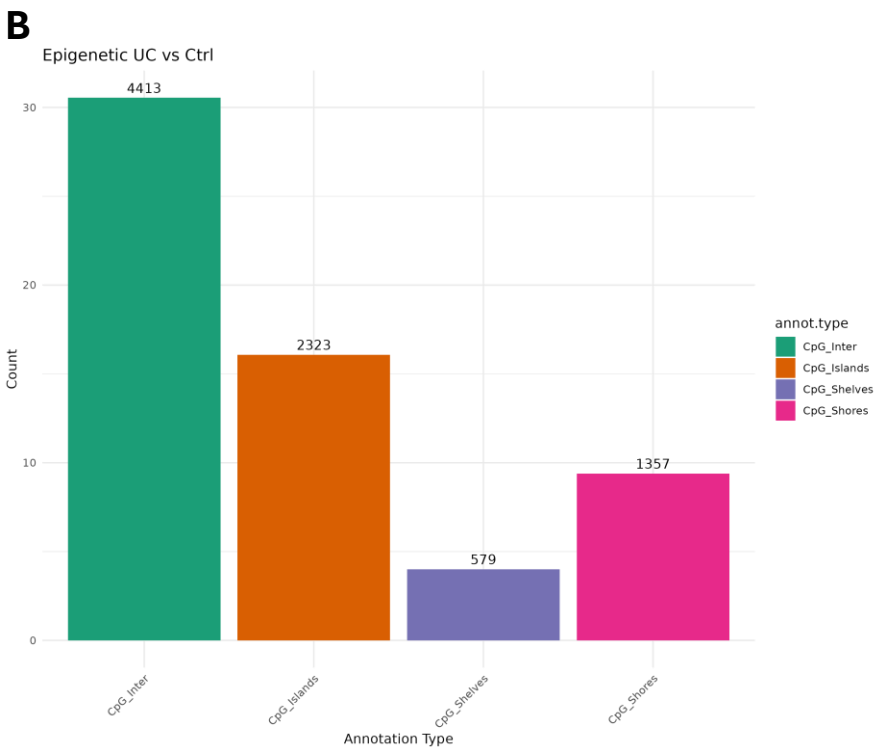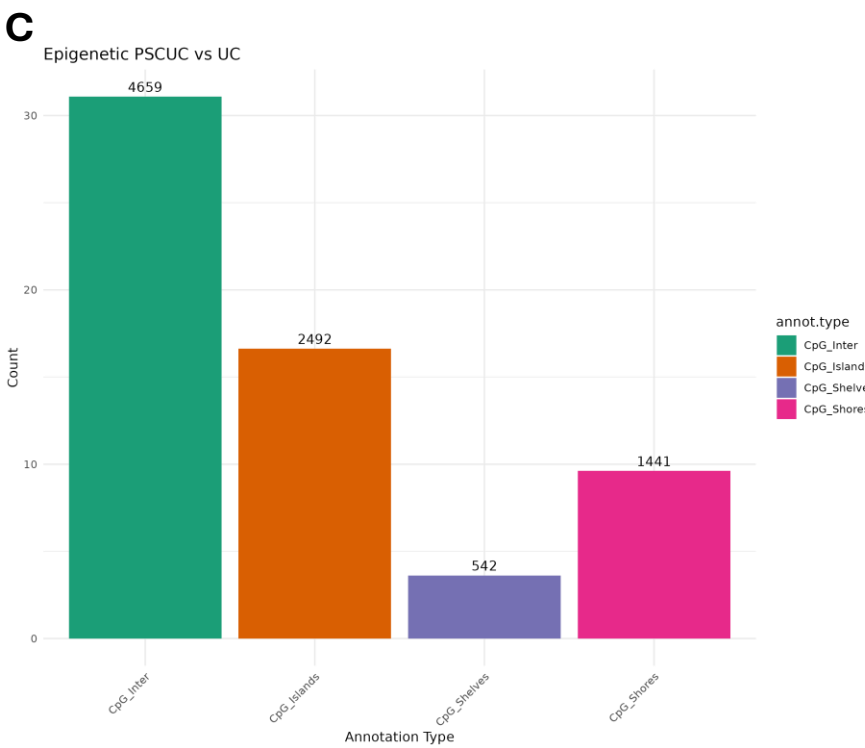

Supplement: Figure A6 [file mmc11.pdf]

# Supplementary figure 7

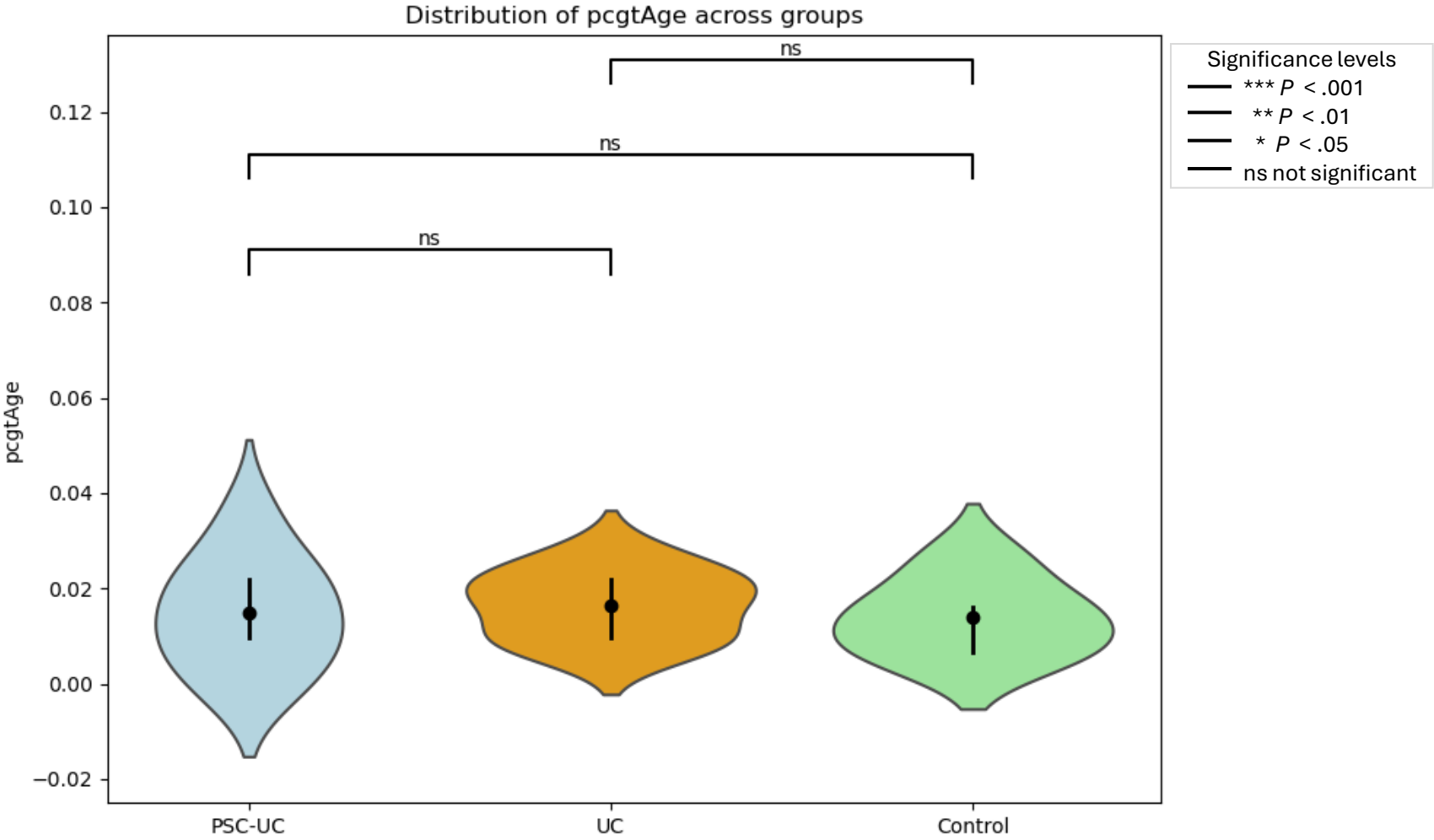

Supplement: Figure A7 [file mmc12.pdf]
