# Supplementary material for: Multiomic Sequencing Reveals Distinctive Gene Expression and Epigenetic Alterations Associated With Primary Sclerosing Cholangitis Development in Treatment-Naïve Pediatric Ulcerative Colitis
Source: Gastro Hep Adv. 2024 Nov 16;4(3):100586. doi: 10.1016/j.gastha.2024.11.002 (PMC11815658; doi:10.1016/j.gastha.2024.11.002)
Supplement: Supplementary Methods [file mmc13.docx]

**Supplementary Methods**

**mRNA sequencing and data analysis**

In brief, the following steps were carried out: Quality Control (FastQC, v0.11.9)^[16]^, adapter and quality trimming (Trim Galore!, v0.6.6)^[17]^, alignment to GRCh38 (STAR, v2.6.1d) ^[18]^, quantification (Salmon, v1.4.0)^[19]^ and deduplication (picard, v2.23.9)^[20]^.

Ultimately, DESeq2 was used to fit the negative binomial model and executed hypothesis testing using the Likelihood Ratio Test or Wald Test[21]. The log2 fold change (FC) ≥0.584 and adjusted p-value ≤0.05 were set to select the differentially expressed genes for each comparison. DEGs with a log2FC ≤-0.584 were considered downregulated genes, while DEGs with a log2FC ≤0.584 were considered upregulated. Principle Component Analysis (PCA) was done with the prcomp function on variance stabilized transformed counts as produced by DESeq2 and visualized using ggplot2.

**Analysis of methylcapture Sequencing Data**

The selected workflow was the bwa-meth profile for alignment, following a previous methylation analysis workflow proposed by the research group, and uses MethylDackel (v0.6.0) to perform methylation calling. Following alignment, using BS-SNPer (v1.1) we called SNPs and generated the .bam files which we used as input for Picard tools CollectHsMetrics (v2.26.3) to perform QC. PCT_SELECTED_BASES was chosen as our main evaluation metric. Using the 10th percentile as a threshold, we removed a total of 2 samples from the PSC-UC group.

Following the initial QC, the differential methylation analysis was conducted using a combination of the R packages RnBeads (v3.16) and DMRcate (v2.14.1) to identify differentially methylated regions (DMRs). A PCA of the data was performed to assess sample clustering, and a second sample from the UC group was removed. The rest of the analysis was carried out with a total of 5 healthy controls, 8 UC patients, and 9 PSC-UC patients. Due to limitations on RnBeads (non-modifiable window size of 5kb for DMR identification), we used the output of an FDR-corrected beta-value matrix from RnBeads as input for DMRcate, where we defined a custom window size of 500 bp by setting the kernel size defining stringency (C) at 2, and the bandwidth defining the smoothing of the window borders (λ) at 1000. Three comparisons were performed corresponding to pediatric patients affected by PSC and UC versus healthy controls, UC patients versus healthy controls, and PSC-UC patients versus UC patients. Using the Stouffer scores derived from DMRcate we defined significant DMRs. A significance threshold was set at -log10(0.05), and regions were classified as hypermethylated or hypomethylated based on whether the -log10 transformed values were > or < than 0, respectively.

Annotation of the identified DMRs was done by a combination of DMRcate and the R package Annotatr (v1.26.0). Two of the cached databases, hg19_genes_intergenic and hg19_basicgenes, were accessed directly through preset shortcuts. To annotate promoters and enhancers, we used two helper functions: build_enhancer_annots() and build_annotations(). These adjustments allowed us to access the databases hg19_enhancers_fantom and hg19_genes_promoters. The default definition of promoters in Annotatr is 1Kb upstream and 100 bp downstream of the transcription start site (TSS). We modified build_annotations() with a custom definition of 2Kb upstream and 500 bp downstream of the TSS. For enhancer annotation, we utilized the function build_enhancer_annots() with the Functional Annotation of the Mammalian Genome 5 (FANTOM5) data (Phase 2). Enhancers are defined as permissive enhancer regions identified by FANTOM5 for the hg19 genome, which includes genomic coordinates annotated as enhancers, based on CAGE data.

We finally carried out an integration analysis of differential gene expression and differential methylation, using the log2FC values from the methylation analysis and the gene expression analysis, to identify genes both up- or down-regulated and hyper- or hypomethylated.

**Histopathological analysis using deep learning**

We employed a deep learning model to explore the differences between cell types in H&E whole slide images (WSI) generated from colonic biopsies of PSC UC and UC patients and controls. We used a pre-trained multitasking learning model “Cerberus”[33] to perform cell-type inference on these H&E WSIs. Cerberus is a single fully convolutional neural network that takes a multi-task learning approach for simultaneous segmentation and classification of nuclei, glands, lumina and different tissue regions. Using a single network with a shared encoder for each segmentation and classification task ensures it learns strong feature representation via transfer learning during training on data from multiple independent sources. This capability of the model to learn from diverse datasets improves the performance accuracy of cell type detection in H&E WSIs, hence this model was chosen over other single-task cell identification models for our application. Furthermore, the training dataset for this model consisted of image regions taken from H&E-stained colon tissue samples, making it equivalate to our WSIs in terms of tissue origin and staining. To achieve nuclei segmentation and classification, Cerberus was trained on the Lizard dataset2 3 containing nearly half a million labelled nuclei where each nucleus is labelled according to the cell it belongs to. The labels it predicts are epithelial, lymphocyte, plasma, neutrophil, eosinophil and connective tissue where connective tissue is a broader category which includes endothelial cells, fibroblasts and muscle cells.

For optimal performance, we pre-processed the WSIs to closely match the attributes of the Cerberus’s training dataset. We used QuPath4 to manually annotate the tissue regions in order to exclude irrelevant regions of the image. The annotated regions were then split into patches of size 448 × 448 pixels at 0.5 µm/pixel. The patches were then fed in a batch size of 9 to the pre-trained Cerberus model running on NVIDIA A100 GPU on a cloud computing platform. The nuclei segmentation and classification portion of the output was isolated and the labelled cells in WSIs were counted through a Python script. The cell count was normalized as the number of cells of a given type with respect to the total number of cells. Statistical comparison was done using Statannotations5 package. Welch’s t-test was performed with Bonferroni correction to compare the means of normalized cell counts of each cell type in PSC UC vs. UC, PSC UC vs controls, and UC vs controls.

**Supplementary Figure Legends**

**Supplementary Figure 1: Principal Component Analysis (PCA)** was generated for **A**. mRNAsequencing in R before carrying out DEGs using DESeq2 package, and **B**. methylation sequencing was generated at an earlier step in the DMRcate pacakage in R. In **A**. mRNAsequencing, each group represented by its respective colours. Healthy control (red dot), PSC-UC (green), and UC (blue). In **B**. methylation sequencing, each group represented by its respective colours. Healthy control (blue dot), PSC-UC (green dot), and UC (red dot).

**Supplementary Figure 2: Flowchart for analysis**. In purple, experimental flowchart. In green, data analysis flowchart of the 30 patients collected (PSC-UC=10, UC=10, Healthy =10). BS-Seq: Bisulphite sequencing. RNA-seq: RNA-sequencing; mRNA: messenger RNA; GO: Gene Ontology; KEGG: Kyoto Encyclopaedia of Genes and Genomes; DEGs: Differentially expressed genes; DMRs: differentially methylated regions; MTRs: Master transcriptional factors.

**Supplementary Figure 3: Comparative histopathological analysis of cell types.** This figure presents six plots per group (PSC-UC, Control, UC), each representing different cell types: neutrophil, epithelial, lymphocyte, plasma, eosinophil, and connective. These plots have been generated as outputs from Cerberus for histopathological analysis. The x-axis for each plot indicates the group names (PSC-UC, Control, UC), and the y-axis represents the respective histopathological measure. While most plots are annotated with "ns" (not significant), indicating no significant differences between the groups, some plots display "**" indicating a degree of significance (p ≤ 1.00e-02). The arrangement allows for a clear comparison of cell type distribution across the groups, highlighting specific instances of significant variation amidst predominantly non-significant findings.

**Supplementary Figure 4:** Identifying genes corresponding to the top two identified master transcriptional regulators along with their transcription factors in UC vs. healthy control. (A) UC vs. healthy control upregulated upset plot showed the top three TFs(highest regulatory score) and the genes they are controlling together. From our input list, 92 genes are controlled by TCF3, RELA, and AR TFs. (B) Gene ontology andpathway of the 92 genes were analysed using DAVID, and the top 15 GO/pathway were plotted. (C) UC vs. healthy control downregulated top three TFs can be seen on the upset plot with 14 overlapping genes. From our input list, 14 genes are controlled byMYOG, PDX1, and SRF TFs. (D) From the GO of the overlapping genes, there were no significant GO/pathway terms. The GO/pathway term was selected with the FDR value <0.05 (blue to red: significant), grey (not significant). y-axis show GO terms and x-axis shows number of hits. “Count”: number of genes that belong to a given gene set.

**Supplementary Figure 5: Identifying genes corresponding to the top two identified master transcriptional regulators along with their transcription factors in PSC-UC vs healthy control.**  (A) PSC-UC vs healthy control upregulated upset plot showed the top three TFs (highest regulatory score) and the genes they are controlling together. From our input list, 366 genes are controlled by RELA, RXRA, and MYOG TFs. (B) Gene ontology of the 366 genes were analysed using DAVID, and the top 15 GO/pathway were plotted. (C) PSC-UC vs healthy control downregulated top three TFs can be seen on the upset plot with 12 overlapping genes. From our input list, 12 genes are controlled by MYOG, MYC, and NFATC1 TFs. (D) From the GO/pathway of the overlapping genes, no GO terms were significant. The GO/pathway term was selected with the FDR value <0.05 (blue to red: significant), grey (not significant). y-axis show GO terms and x-axis shows number of hits. “Count”: number of genes that belong to a given gene set.

**Supplementary Figure 6:** **Annotated epigenetic domains for DMRs per comparison obtained from DMRcate differential methylation analysis.** R package annotatr was used to annotate the regions. In the y-axis we have the final count of annotation and in the x-axis the main annotation groups as defined in annotatr documentation. These categories are: CpG islands, shores (2Kb up/downstream from the border of a CpG island), shelves (located another 2Kb up/downstream of the farthest limits of shores) and Open-Sea or Inter (defined as the remaining regions in-between).

**Supplementary Figure 7: Distribution of pcgtAge across comparisons.** This figure illustrates the distribution of pcgtAge across three groups: PSCUC, UC, and Control, using the epiTOC2 method to generate the epigenetic age per group. The data is visualized with three violin plots, each representing one of the groups. The x-axis is labeled with the group names (PSCUC, UC, Control), and the y-axis is labeled "pcgtAge." Each violin plot has an annotation indicating "ns" (not significant), meaning the statistical analysis found no significant differences in pcgtAge between the groups. The violin plots are arranged side by side to facilitate easy comparison, highlighting the lack of significant variation in epigenetic age among the groups.

**Supplementary Tables**

**Supplementary Table 1: Differentially expressed genes (DEGs) identified in all comparisons.** In PSC-UC versus healthy control we identified 1,180 genes up-regulated and 892 downregulated. When UC versus healthy control was compared, we identified 249 genes up-regulated and 149 down-regulated. Finally, when PSC-UC versus UC was compared we identified 9 upregulated and 5 downregulated. Using cut of value of LogFC ± 0.584 and adjp-value of < 0.05.

**Supplementary Table 2: Transcription Factor Enrichment Analysis table of all the identified DEGs.** The identified TFs are linked to the site models in the composite module identified by the workflow. Each row shows details for TF, including its Ensembl gene ID, gene symbol, gene description and biological species of the corresponding TF. The column Site model ID shows the identifier of the PWM associated with this TF. The columns Yes density per 1000bp and No density per 1000bp show the number of matches normalized per 1000 bp length for the sequences in the input Yes set and input No set, respectively.

**Supplementary Table 3:** **Transcription factors of the predicted enhancer model** p**otentially regulating the differentially expressed**. Yes-No ratio is the ratio between frequencies of the sites in Yes sequences versus No sequences. It describes the level of the enrichment of binding sites for the indicated TF in the regulatory target regions. Regulatory score is the measure of involvement of the given TF in the controlling of expression of genes that encode master regulators presented below (through positive feedback loops).

**Supplementary Table 4**: **Master regulators potentially regulating the upregulated differentially expressed.** Total rank is the sum of the ranks of the master molecules sorted by keynode score, CMA score. The score value of each master regulatory molecule indicates its connectivity in the database and presence of input list molecules in its network. Higher Score values signify stronger connections and more "Hits" from the input list. Only molecules with Score > 0.2 are displayed by default in the output.

**Supplementary Table 2: Transcription Factor Enrichment Analysis table of all the identified DEGs.** The table includes the genomic location of each differentially methylated region (DMR) with the first three columns indicating the chromosome (chrom), start position (start), and end position (end) of the DMR. The width column shows the size of the DMR in base pairs. The min_smoothed_fdr column provides the minimum smoothed false discovery rate for each DMR, indicating its statistical significance. Stouffer and HMFDR columns display the combined p-value using Stouffer's method and the harmonic mean false discovery rate, respectively, offering additional measures of significance. The meandiff column shows the mean difference in methylation levels between groups. The overlapping.genes column lists genes that overlap with each DMR. The annot.type column specifies the type of genomic feature associated with each DMR, while the methylation column indicates whether the DMR is hypermethylated or hypomethylated. The annot.chrom, annot.start, annot.end, and annot.width columns detail the chromosome, start and end positions, and width of the annotated feature, respectively, with annot.symbol providing the symbol of the annotated gene or feature, obtained with the package annotatr.
